# Rising Incidence and Mortality of Early-Onset Colorectal Cancer in Young Cohorts Associated with Delayed Diagnosis †

**DOI:** 10.3390/cancers17091500

**Published:** 2025-04-29

**Authors:** Yazan Abboud, Anand Shah, Madison Fraser, Eric M. Montminy, Chun-Wei Pan, Kaveh Hajifathalian, Paul J. Gaglio, Ahmed Al-Khazraji

**Affiliations:** 1 Department of Internal Medicine, Rutgers New Jersey Medical School, Newark, NJ 07103, USA; ass258@njms.rutgers.edu (A.S.); mf1124@njms.rutgers.edu (M.F.); 2 Division of Gastroenterology and Hepatology, University of Chicago, Chicago, IL 60637, USA; eric.montminy@bsd.uchicago.edu; 3 Department of Internal Medicine, John H. Stroger Jr. Hospital of Cook County, Chicago, IL 60612, USA; chunwei.pan@cookcountyhealth.org; 4 Division of Gastroenterology and Hepatology, Rutgers New Jersey Medical School, Newark, NJ 07103, USA; kh852@njms.rutgers.edu (K.H.); pjg47@njms.rutgers.edu (P.J.G.); aa2758@njms.rutgers.edu (A.A.-K.)

**Keywords:** early-onset colorectal cancer, incidence, mortality, colorectal cancer screening, epidemiology

## Abstract

**Background and Aims**: Prior data showed an increasing incidence of early-onset colorectal cancer (EO-CRC) in the US. However, there are limited comprehensive data on recent EO-CRC incidence and mortality per different age cohorts and tumor characteristics. We aimed to evaluate EO-CRC incidence and mortality in different populations. **Methods**: Age-adjusted EO-CRC incidence rates were calculated from the USCS database between 2001 and 2021. Age-adjusted EO-CRC mortality rates were calculated from the NCHS database between 2000 and 2022 and the SEER database between 2004 and 2021. The age groups were 20–44 years and 45–54 years. Tumors were categorized by anatomical location (right, transverse, left, and proximal) and stage at diagnosis (early and late). The annual and average annual percentage changes (AAPC) were estimated using joinpoint regression. Age-specific pairwise comparison was conducted. **Results**: A total of 474,601 patients were diagnosed with EO-CRC between 2001 and 2021. The EO-CRC incidence increased in patients aged 20–44 years faster than in those aged 45–54 years (AAPC = 1.51 vs. 0.73; AAPC difference = 0.78, *p* = 0.001). This was most notable in proximal colon tumors (AAPC difference = 0.88, *p* < 0.001). While the incidence of early-stage tumors decreased in recent years across all anatomical locations, late-stage tumors increased, mostly in the proximal colon (AAPC = 2.44). A total of 147,026 patients died from EO-CRC between 2000 and 2022. Mortality increased in patients aged 20–44 years faster than in patients aged 45–54 years, who had a stable trend (AAPC difference = 0.85, *p* < 0.001). The increase in mortality was noted in both early- and late-stage tumors. **Conclusions**: Our study provides epidemiologic evidence showing an alarming increase in EO-CRC incidence and mortality among patients aged 20–44 years compared to those aged 45–54 years, which is associated with delayed diagnosis, and mostly in proximal colon tumors. Expanding screening efforts to younger populations and addressing healthcare disparities are essential for improving early detection and outcomes for younger patients.

## Simple Summary

While there is emerging evidence showing increasing incidence of early-onset colorectal cancer (EO-CRC) in the US, there remain gaps in the literature on recent trends of EO-CRC incidence and mortality stratified by age cohorts and tumor anatomical locations. The current study is a time-trend analysis utilizing three national databases, United States Cancer Statistics, National Center for Health Statistics, and Surveillance, Epidemiology, and End Results, to evaluate the incidence and mortality of EO-CRC among cohorts aged 20–44 years and those aged 45–54 years, and in different anatomical parts of the colon. We demonstrate increasing incidence and mortality of EO-CRC among the younger cohort, which was found to be associated with delayed diagnosis and mostly arising from proximal colon tumors. Our study highlights recent trends of EO-CRC burden, reflecting on the importance of considering expanding screening efforts to younger cohorts. Future efforts should be tailored toward addressing healthcare disparities with the goal of improving early detection and, ultimately, the survival of younger adults with colorectal cancer.

## 1. Background

Colorectal cancer is the third most diagnosed cancer globally and the second cause of cancer-related deaths worldwide [1]. Despite improvements in detection, there exists an alarming increase in early-onset colorectal cancer (EO-CRC) incidence over the past decades [2], with early onset defined as colon or rectal cancer occurring in men or women under age 50 [3]. This increase in younger individuals prompted medical societies to change the initial screening age recommendation to 45 in 2021 [4]. Despite the increasing mortality of EO-CRC in the US [5], there remain many unanswered questions about the characteristics and outcomes of populations most at risk.

Most EO-CRC tumors occur in the left colon [6,7]. This is in part because left-sided malignancies tend to present with more symptoms prompting diagnostic evaluations compared to right-sided colon tumors [8,9]. Data are limited on the incidence and mortality of EO-CRC in the US in different anatomical locations and stages at diagnosis. Moreover, evidence comparing the rise in EO-CRC incidence among different age groups is scarce.

To address these gaps, we utilized several national representative databases to examine the trend of EO-CRC over the past two decades in multiple capacities. This included comparisons of incidence, patient demographics, tumor characteristics (anatomical location, staging), and mortality rates.

## 2. Materials and Methods

### 2.1. Study Design

This is a retrospective cohort study of publicly available epidemiological data. Cohort data were obtained via the nationally available databases of United States Cancer Statistics (USCS), National Center for Health Statistics (NCHS), and Surveillance, Epidemiology, and End Results (SEER).

### 2.2. Data Collection

#### 2.2.1. USCS Database

Incidence rates for EO-CRC between 2001 and 2021 were obtained from the USCS database, which covers nearly 98% of the US population [10]. USCS data are sourced from two national programs: the CDC’s National Program of Cancer Registries (NPCR), and the NCI’s SEER. After the data are collected, several processes are performed to ensure the maintenance of high-quality data and standardization [11].

#### 2.2.2. NCHS Database

Mortality rates of EO-CRC between 2000 and 2022 were obtained from the NCHS database, which covers nearly 100% of the US population [12]. NCHS data are sourced from the National Vital Statistics System, which collects data on deaths from state vital registries. The data are automated, standardized, and continuously reviewed upon their collection.

#### 2.2.3. SEER Database

Incidence-based mortality (IBM) rates between 2004 and 2021 were obtained from the SEER 22 database, which covers nearly 42% of the US population [13]. SEER mortality data are also sourced from the National Vital Statistics System.

While overall mortality was evaluated using the NCHS database, stage-specific mortality was evaluated using the SEER 22 database.

All three databases used in this study, USCS, NCHS, and SEER, undergo rigorous quality assurance and standardization protocols prior to public release [14]. The SEER and USCS programs maintain consistent data coding practices over time by utilizing standardized ICD-O-3 histology and site codes, as well as stable staging definitions during the study period. As a result, the observed trends are unlikely to be significantly affected by changes in coding or classification systems.

### 2.3. Definitions

EO-CRC incidence and mortality rates were defined as the number of patients aged 20–54 years who were diagnosed and whose death was attributed to CRC per 100,000 population in a calendar year, respectively. IBM rates were defined as the number of patients aged 20–54 years with known incidence data whose death was attributed to CRC. Time trends were reported as annual percentage change (APC) and average APC (AAPC). Younger adults were defined as adults aged 20–54 years [2,15,16], and were categorized into two age cohorts: 20–44 and 45–54 years.

We defined EO-CRC as colorectal cancer in adults aged 20 to 54 years, based on previous studies using SEER and national databases. This definition helps identify trends in younger patients not typically included in screening efforts before the 2021 USPSTF guideline change. We divided the cohort into two age groups: 20 to 44 years, representing a predominantly unscreened population, and 45 to 54 years, which is partially screened due to screening recommendations established in the late 1990s. This approach aligns with existing epidemiologic literature on EO-CRC trends [15,16]. Anatomical locations were the right-sided colon (cecum, ascending colon, and hepatic flexure), transverse colon, left-sided colon (splenic flexure and descending colon), and proximal colon (sigmoid colon, rectosigmoid junction, and rectum). Stages at diagnosis were early stage (localized and in situ) and late stage (regional and distant). Given that most CRCs are adenocarcinomas, which also have the highest burden of mortality, the EO-CRC cancers in our analysis were only adenocarcinoma cases with malignant behavior (Supplementary Materials) [2,16].

### 2.4. Statistical Analysis

EO-CRC incidence, mortality, and IBM rates were calculated and age-adjusted to the standard 2000 US population using SEER*Stat software (v.8.4.3, NCI, Bethesda, MD, USA). This was done in all three databases used in our analysis, in order to maintain direct standardization. The rates were categorized by age cohort, tumor anatomical location, and stage at diagnosis. Rate ratios were calculated and compared using the Tiwari method [17]. APC and AAPC were estimated via Joinpoint Regression software (v.5.2.0.0, NCI) using the weighted Bayesian Information Criterion “BIC” method [18,19,20]. The minimum number of joinpoints used in our analysis was 0, and the maximum number was 3, as recommended by the Joinpoint Regression software. Pairwise comparison between the age cohorts was conducted using the tests of coincidence and parallelism (*p*-value < 0.05) [21]. Lastly, we conducted two sensitivity analyses. The first was after stratifying patients into 5-year interval age cohorts to further investigate the trends between different age cohorts. The second was replicating the initial analysis but including all histopathological subtypes of EO-CRC.

## 3. Results

### 3.1. Incidence Rates and Time Trends of EO-CRC

There were 474,601 patients aged 20–54 years who were diagnosed with EO-CRC between 2001 and 2021 in the USCS database. Most were males (55.2%), of Non-Hispanic-White race/ethnicity (67.3%), and aged 50–54 years (46.2%) (Supplementary Table S1 and Supplementary Figure S1). The incidence was significantly higher in males (16.5/100,000) compared to females (13.3/10,000), with a rate ratio of 0.80 (*p* < 0.001). The highest incidence was noted in proximal colon tumors [9.3/100,000; (62.9%)], late-stage tumors [9.7/100,000; (64.9%)], and in the US Southern region [15.9/100,000; (39.4%)]. The incidence was increasing in patients aged 20–44 years at a significantly faster pace compared to patients aged 45–54 years (AAPC = 1.51 vs. 0.73; AAPC difference = 0.78, *p* = 0.001) with non-identical non-parallel data (*p*-values < 0.01). Joinpoint regression revealed that the 45–54-year cohort experienced a relatively stable trend in the early years, followed by a steeper rise in incidence during the latter part of the study period, as reflected in the segmented APCs. In contrast, the 20–44-year group showed a more consistent linear increase across the entire time period.

### 3.2. Age-Specific Incidence Rates and Time Trends of EO-CRC in Different Anatomical Locations

**Figure 1:**
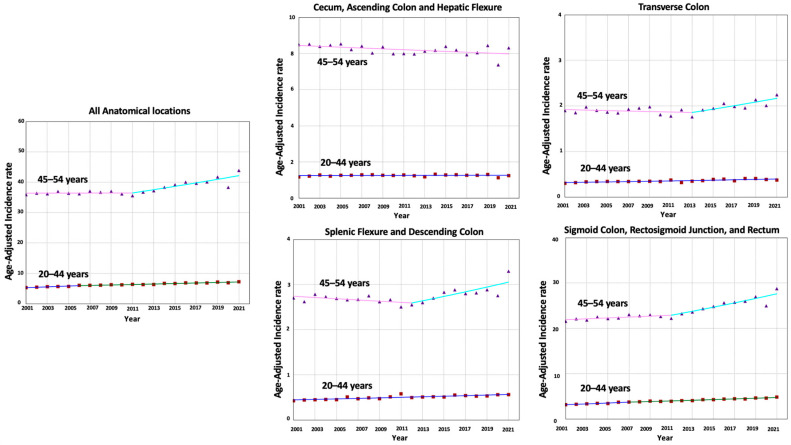
Time trends and incidence rates for EO-CRC categorized by tumor anatomical location in different age cohorts.

For right-sided colon tumors (100,517 patients), incidence decreased in adults aged 45–54 years (AAPC = −0.28, *p* = 0.01) but not in those aged 20–44 years (AAPC = 0.07, *p* = 0.63). For transverse colon tumors (25,027 patients), incidence increased in adults aged 20–44 and 45–54 years (AAPC= 1.08 vs. 0.61; AAPC difference = 0.47, *p* = 0.16). For left-sided colon tumors (35,260 patients), incidence increased in adults aged 20–44 years while remaining stable in those aged 45–54 years (AAPC= 1.21 vs. 0.54; AAPC difference= 0.67, *p* = 0.05). Lastly, for proximal colon tumors (298,417 patients), incidence increased in both age cohorts, with a faster pace in adults aged 20–44 (AAPC= 2.04 vs. 1.16; AAPC difference = 0.88, *p* < 0.001), suggesting that the disparity between age cohorts is largely driven by proximal colon tumors (Table 1 and Figure 1).

### 3.3. Stage-Specific Incidence Rates and Time Trends of EO-CRC in Different Anatomical Locations

The incidence of EO-CRC increased between 2001 and 2021 in all anatomical locations except right-sided colon tumors, which remained stable (AAPC = −0.18; *p* = 0.13) (Table 2).

**Figure 2:**
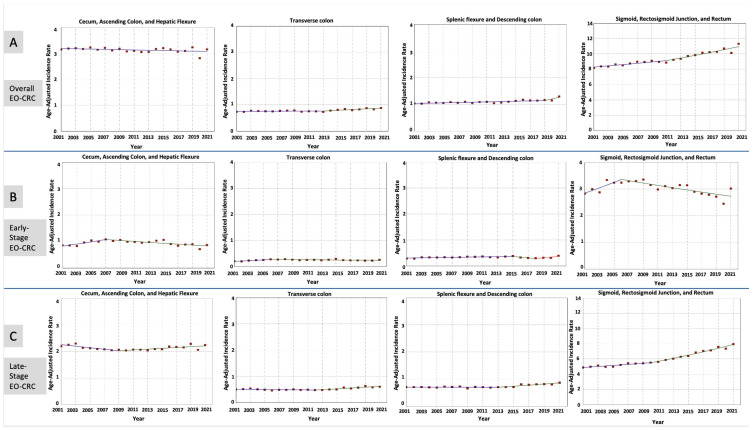
Time trends and incidence rates for EO-CRC categorized by tumor anatomical location and stage at diagnosis: (**A**) All Stages of EO-CRC, (**B**) Early-Stage EO-CRC, (**C**) Late-Stage EO-CRC.

For early-stage tumors, incidence rates initially increased in the first decade, followed by a decline in recent years across all anatomical locations. In contrast, late-stage tumors experienced a consistent rise in incidence during the second decade of our study across all different anatomical locations. This was most notable in proximal colon tumors, which showed a significant rise throughout the study period (AAPC = 2.44; *p* < 0.001) (Table 2 and Figure 2).

### 3.4. Mortality Rates and Time Trends of EO-CRC

Between 2000 and 2022, there were 147,026 deaths attributed to EO-CRC in the NCHS database. Most of the patients were males (56.6%), of Non-Hispanic-White race/ethnicity (65.9%), and aged 50–54 years (45.7%) (Supplementary Table S2 and Figure S2). The mortality rate was significantly higher in males (4.8/100,000) than in females (3.6/100,000), with a rate ratio of 0.76 (*p* < 0.001). The highest mortality burden was noted in late-stage tumors [3.3/100,000; (79.2%)], and in the US Southern region [4.8/100,000; (41.8%)].

Mortality rates increased in patients aged 20–44 years (AAPC = 0.93; *p* < 0.001) at a much faster pace than in patients aged 45–54 years, whose trend remained stable (AAPC = 0.09; *p* = 0.58), with an AAPC difference of 0.85 (*p* < 0.001) and non-identical non-parallel data (*p*-values < 0.001).

**Figure 3:**
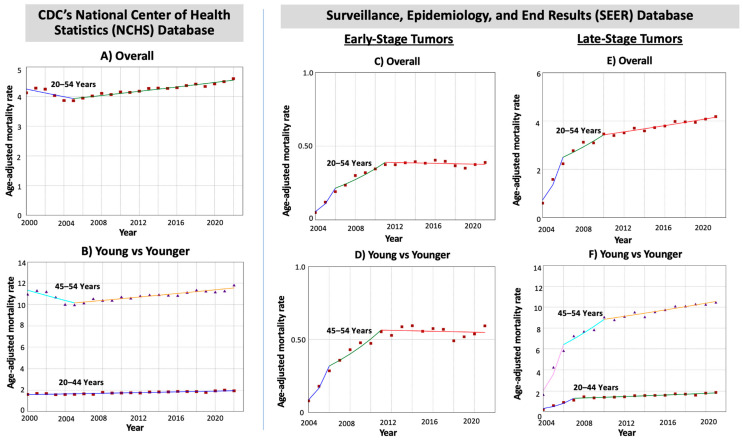
Time trends and mortality rates for EO-CRC in different age cohorts categorized by tumor stage at diagnosis.

For early-stage tumors (3528 deaths; 7.5%), mortality increased in adults aged 45–54 years between 2004 and 2011 and stabilized afterward. For late-stage tumors (37,472 deaths; 79.2%), mortality increased throughout the study period (AAPC = 10.69), and this was seen in both age cohorts: 45–54 years (AAPC = 10.15) and 20–44 years (AAPC = 10.66) (Table 3 and Figure 3).

### 3.5. Sensitivity Analyses

Incidence rates were increasing in all 5-year-interval age cohorts except those aged 20–24 years. The most rapid increase was in patients aged 35–39 years (AAPC = 1.59; *p* < 0.001), followed by those aged 40–44 years (AAPC = 1.42; *p* < 0.001) (Supplementary Table S3 and Figure S3).

The second sensitivity analysis, including all histopathological subtypes of EO-CRC, showed similar results to the initial analysis, including only adenocarcinomas (Supplementary Tables S4–S7 and Figures S4–S7).

## 4. Discussion

Our study, which comprehensively evaluates the burden of EO-CRC in the US, demonstrates that younger cohorts have been experiencing a higher increase in EO-CRC incidence and mortality over the past two decades, associated with delayed diagnosis. This alarming trend was mostly noted in proximal tumors of the colon that were diagnosed at advanced stages. This rise was noted in early-onset adenocarcinomas and EO-CRC of all histopathological subtypes, with the sharpest uptick in patients aged 35–39 and 40–44 years.

Prior literature evaluating EO-CRC incidence in different age cohorts is scarce. The definitions of EO-CRC vary in the literature, with some studies using a cutoff age of 49 years. However, our selection of a 20–54-year age range aligns with several recent SEER-based analyses and allows for a more comprehensive assessment of epidemiological trends among younger populations [15,16]. Additionally, we conducted sensitivity analyses utilizing 5-year age cohorts to further clarify these trends. This included a specific evaluation of patients aged 35–39 and 40–44 years, where we observed the steepest rise in incidence (Supplementary Table S3). In the US, Shah et al. conducted a non-comparative observational study showing an increasing incidence of EO-CRC between 2001 and 2017, with the steepest increases seen in those aged 20–24 years [22]. This trend was also seen outside of the US. A study across 20 European countries between 1990 and 2016 found that CRC incidence began rising earlier in the 30–39 age group, with the most significant increase in patients aged 20–39 [23]. While these studies somewhat correlate with our data, we add to the literature by providing comparative, representative, and recent data from the US showing that the most significant increase was seen in patients aged 20–44 years, while further stratifying the trends by tumor location.

Evolving screening recommendations and diagnostic testing behavior likely influenced trends in EO-CRC detection in our study. CRC screening for average-risk individuals aged ≥50 was first recommended in 1996 and reinforced in 2002 by the USPSTF, leading to increased screening uptake in the 50–54 age group over the past two decades. Importantly, the 45–54-year-old age group likely represents a mixed cohort of partially screened and unscreened individuals, particularly in the earlier years of our study, when CRC screening was not yet widely adopted in this age group.

While current guidelines shifted to start screening average-risk individuals at the age of 45, younger adults may undergo colonic examination for a variety of reasons, including gastrointestinal bleeding, inflammatory bowel disease, irritable bowel syndrome, hereditary syndromes, or a family history of CRC. A large study involving 84 gastroenterology practices in the US evaluating 1,372,838 colonoscopy reports found nearly a three-fold increase in colonoscopy rates between 2000 and 2011 [24]. Moreover, a large employer-based claims database study showed a 30% increase in the rates of colonoscopies in younger adults between 2001 and 2009 [25]. Furthermore, after the adoption of colonoscopy as the preferred screening method and Medicare’s inclusion of this modality, many ambulatory surgery centers opened in the late 1990s and early 2000s, which likely contributed to increasing endoscopy capacity and lowering the threshold for performing colonoscopies in younger patients [26,27]. However, one would expect increased detection and resection of pre-cancerous lesions with the increased utilization of colonoscopies, which should lead to a decline in the rate of CRC. However, the persistent and accelerated increase in late-stage proximal tumors, particularly among unscreened younger adults, suggests that improvements in diagnostic access alone do not fully account for these trends. Thus, there must be risk factors disproportionally affecting younger cohorts, such as obesity, diabetes, tobacco and alcohol consumption, physical inactivity, and increased consumption of animal fats [28,29,30], especially in countries with a high human development index [31].

Our findings align with other prior data indicating that young CRC patients predominantly have left-sided tumors [6]. A national cancer registry analysis in England demonstrated that the fastest rise in CRC incidence among those aged 20–39 years was in the right colon [32]. We provide evidence from the US showing a more rapid increase in EO-CRC in proximal colon tumors. The increased incidence of left-sided EO-CRC can be attributed to a combination of genetic, molecular, and environmental factors. There is evidence suggesting that left-sided malignancies are more often associated with unique molecular features, such as higher rates of mutations in the TP53 and PTEN genes, which are less common in right-sided malignancies and may lead to more aggressive tumor behavior [33]. Additionally, right-sided colon lesions are often asymptomatic due to the larger luminal diameter and the possibility of decompression via the cecum and ileocecal valve mechanism, which lowers the risk of intestinal obstruction. In addition, when taking into account the increasing incidence of IBD [34], a higher incidence of rectal tumors was seen in patients with ulcerative colitis, while no such difference between left- and right-sided tumors was seen in Crohn’s [35]. Thus, this is less likely to explain the more pronounced increase noted in proximal colonic tumors. A single-center study of 269 patients with EO-CRC showed that tumors were more likely to be diagnosed in the left colon and at a late stage [36]. Future studies are warranted to investigate risk factors associated with EO-CRC risk in different anatomical locations.

A growing body of literature is showing that EO-CRC differs from late-onset CRC and tends to be more aggressive and present at a later stage. A prospective international cohort study showed that EO-CRC patients have more advanced disease at the time of diagnosis compared to late-onset CRC [37]. From a histological perspective, younger patients present with a higher prevalence of mucinous or poorly differentiated tumors, including signet ring tumors and venous, perineural, and lymphovascular invasion, which are associated with poor survival and prognosis [37,38]. Furthermore, a single-center study showed that the majority of EO-CRCs are sporadic, non-Lynch, and microsatellite stable tumors compared to late-onset CRCs, which are more aggressive, with poorer outcomes, suggesting a potentially distinct carcinogenic pathway [39]. Another study demonstrated that EO-CRC is often not linked to hereditary syndromes but shows a distinct predilection for the distal colon with aggressive histologic features [38]. Additionally, younger patients usually present with late-stage tumors, likely due to the absence of routine screening and the misinterpretation of symptoms as less serious conditions such as hemorrhoids or irritable bowel syndrome, which often leads to delayed diagnosis. This may explain, in part, the increasing rates of late-stage disease in younger cohorts that were observed in our study. The initial rise and subsequent decline in early-stage tumors observed in our study may be attributed to enhanced screening practices, which likely improved early detection earlier in the study period. However, the stabilization or decline in early-stage tumor incidence in recent years may indicate a saturation effect, where the most at-risk populations are being screened, leaving a gap in detection among those who may not have been previously considered high-risk [40,41]. Our findings suggest that initial screening at age 45 may not be early enough to capture the rising incidence of EO-CRC, especially given the trends in late-stage diagnosis.

Overall mortality rates from CRC have been declining in recent years, and this was attributed to improved screening modalities and their utilization, and treatments [42]. Our study highlights the increase in mortality observed among the younger cohort, aged 20–45 years, which was significantly faster, and occurred in both early- and late-stage tumors. These findings hold important implications, prompting the re-evaluation of current management guidelines. While there are data showing the benefits of neoadjuvant chemotherapy in EO-CRC [43], surgical resection remains the standard treatment, with adjuvant chemotherapy in chosen cases [44]. Our findings prompt investigations of the outcomes of current management modalities of EO-CRC, especially the role of neoadjuvant chemotherapy in these patients.

Our analysis of EO-CRC mortality exposed a significant parallel between younger patient age and higher mortality. This could be explained by the increase in incidence of late-stage tumors. Despite the fact that younger individuals have better survival compared to their older counterparts [45], our findings are alarming, showing that younger individuals are developing EO-CRC both more frequently and with worsening mortality. Healthcare access disparities, delays in seeking medical care, and potential biological differences in tumor progression between different age cohorts may contribute to the increased mortality of early- and late-stage EO-CRC [38,42,43,44,45,46]. Having said that, the sharp increase in stage-specific mortality rates shown in Panels C–F of Figure 3 of the current manuscript during the first few years may be partly due to improvements in registry data and the expanded coverage of the SEER 22 registry, which grew to include about 42% of the US population. This inclusion of more geographic regions likely influenced the early rise in reported mortality rates for stage-specific cases and should be considered when interpreting the initial trends. Our findings suggest potential flaws in our current system of CRC screening. Therefore, the goal of this study is not only to serve as a reminder for clinicians to keep the suspicion of EO-CRC amongst younger patients elevated, but also to be a catalyst for further research into both the potential etiologies and optimal screening guidelines for EO-CRC.

The strengths of our study include the comprehensive recent analysis utilizing three representative national databases, providing the first comparative analysis of incidence and mortality trends between different age cohorts, and highlighting stark disparities affecting the cohort aged 20–44 years. Moreover, we provide data by tumor anatomical location and stage at diagnosis, along with disease burden in different demographics and regions. This is informative given the variation in pathophysiology, management, and prognosis between tumors in different anatomical locations and stages. Furthermore, to account for the emerging rise in colorectal neuroendocrine tumors and any potential miscoding of adenocarcinomas, we performed sensitivity analyses including all histopathological subtypes of EO-CRC and found similar results to the initial analysis of adenocarcinomas. Additionally, we used joinpoint regression and the modified BIC method given their utility in large databases involving large populations [18,19]. Limitations of our study include the unavailability of granular data to assess for risk factors associated with EO-CRC risk, such as family history, smoking history, and other patient co-morbidities. Another limitation includes the possibility of losing records or miscoding in large databases such as the ones we used. Additionally, the use of multiple large databases employed for the statistical analysis of this study allows for a potential lack of standardization, given the differences in data collection and records between different organizations. We also recognize that gradual improvements in data completeness, reporting practices, and coding accuracy over time, especially in the earlier years of our study, may partly account for the observed changes in incidence-based mortality trends.

## 5. Conclusions

In conclusion, our study demonstrates alarming epidemiological evidence of increasing EO-CRC incidence and mortality among adults aged 20–44 years, occurring at a faster pace compared to patients aged 45–54 years, associated with delayed diagnosis. This trend was most pronounced in proximal colon tumors and late-stage malignancies and was driven by adenocarcinomas, especially in younger cohorts. Our findings hold important healthcare implications, highlighting the need to re-evaluate existing CRC screening guidelines. Expanding screening efforts to younger populations, raising awareness of EO-CRC symptoms, and addressing healthcare disparities are critical steps toward improving outcomes for younger CRC patients.

## Figures and Tables

**Table 1:** Time trends of EO-CRC incidence rates between 2001 and 2021 categorized by tumor Anatomical location in different age cohorts. ^a^ Count numbers followed by their percentages from the total cases of EO-CRC. ^b^ Tests whether trends were identical. A significant *p*-value indicates that the trends were not identical. ^c^ Tests whether trends were parallel. A significant *p*-value indicates that the trends were not parallel. * Implies statistical significance.

Age Cohort (Years)	Early-Onset CRC Number of Patients (N = 474,601) ^a^	Trends			Age-Specific AAPC Difference (95% CI)	Pairwise Comparison *p*-Values		
		Time Period	APC (95% CI)	AAPC (95% CI)		Age-Specific AAPC Difference	Test of Coincidence ^b^	Test of Parallelism ^c^
All Anatomical Locations								
20–44 years	129,938 (27.4%)	2001–2006	2.35 * (1.30 to 3.42)	1.51 * (1.24 to 1.79)	0.78 * (0.30 to 1.25)	0.001	<0.001	0.001
		2006–2021	1.23 * (1.05 to 1.42)					
45–54 years	344,663 (72.6%)	2001–2011	0.01 (−0.59 to 0.62)	0.73 * (0.35 to 1.13)				
		2011–2021	1.46 * (0.87 to 2.05)					
Cecum, Ascending Colon, and Hepatic Flexure								
20–44 years	25,772 (5.4%)	2001–2021	0.07 (−0.22 to 0.36)	0.07 (−0.22 to 0.36)	0.35 * (0.01 to 0.70)	0.04	<0.001	0.03
45–54 years	74,745 (15.7%)	2001–2021	−0.28 * (−0.51 to −0.06)	−0.28 * (−0.51 to −0.06)				
Transverse Colon								
20–44 years	7373 (1.6%)	2001–2021	1.08 * (0.73 to 1.43)	1.08 * (0.73 to 1.43)	0.47 (−0.19 to 1.13)	0.16	<0.001	0.05
45–54 years	17,654 (3.7%)	2001–2013	−0.28 (−0.94 to 0.38)	0.61 * (0.04 to 1.18)				
		2013–2021	1.95 * (0.77 to 3.16)					
Splenic Flexure and Descending Colon								
20–44 years	10,364 (2.2%)	2001–2021	1.21 * (0.85 to 1.57)	1.21 * (0.85 to 1.57)	0.67 (−0.01 to 1.34)	0.05	<0.001	0.004
45–54 years	24,896 (5.2%)	2001–2012	−0.51 (−1.29 to 0.28)	0.54 (−0.04 to 1.13)				
		2012–2021	1.84 * (0.80 to 2.88)					
Sigmoid Colon, Rectosigmoid, and Rectum								
20–44 years	81,825 (17.2%)	2001–2007	2.73 * (1.82 to 3.64)	2.04 * (1.75 to 2.34)	0.88 * (0.39 to 1.38)	<0.001	<0.001	<0.001
		2007–2021	1.75 * (1.53 to 1.98)					
45–54 years	216,592 (45.6%)	2001–2011	0.43 (−0.19 to 1.06)	1.16 * (0.76 to 1.56)				
		2011–2021	1.90 * (1.31 to 2.49)					

**Table 2:** Time trends of EO-CRC incidence rates between 2001 and 2021 categorized by tumor anatomical location and stage at diagnosis. ^a^ Count numbers followed by their percentages from the total cases of EO-CRC. * Implies statistical significance.

Tumor Anatomical Location	Early-Onset CRC Number of Patients (N = 474,601) ^a^	Trends				
		Time Period	APC (95% CI)	*p*-Value	AAPC (95% CI)	*p*-Value
All Stages Combined						
Cecum, Ascending Colon, and Hepatic Flexure	100,517 (21.2%)	2001–2021	−0.18 (−0.44 to 0.07)	0.13	−0.18 (−0.44 to 0.07)	0.13
Transverse Colon	25,027 (5.3%)	2001–2013	0.13 (−2.78 to 0.98)	0.93	0.76 * (0.36 to 1.15)	0.001
		2013–2021	1.72 * (0.57 to 5.81)	0.03		
Splenic Flexure and Descending Colon	35,260 (7.4%)	2001–2019	0.55 (−0.33 to 0.86)	0.08	1.02 * (0.60 to 1.24)	<0.001
		2019–2021	5.27 * (0.79 to 7.68)	<0.001		
Sigmoid, Rectosigmoid, and Rectum	298,417 (62.9%)	2001–2011	0.97 (−1.24 to 1.58)	0.20	1.41 * (1.18 to 1.65)	<0.001
		2011–2021	1.85 * (1.35 to 3.86)	0.02		
Early-Stage Tumors						
Cecum, Ascending Colon, and Hepatic Flexure	30,838 (6.5%)	2001–2007	4.53 * (1.85 to 11.62)	<0.001	0.06 (−0.59 to 0.88)	0.85
		2007–2021	−1.79 * (−2.97 to −0.96)	<0.001		
Transverse Colon	7905 (1.7%)	2001–2006	6.84 * (2.85 to 18.52)	<0.001	0.95 * (0.16 to 1.89)	0.02
		2006–2021	−0.94 * (−2.19 to −0.17)	0.02		
Splenic Flexure and Descending Colon	10,932 (2.3%)	2001–2015	1.31 * (0.68 to 2.28)	0.01	0.98 * (0.37 to 1.55)	0.009
		2015–2018	−7.91 * (−11.46 to −2.27)	0.01		
		2018–2021	9.04 * (2.86 to 19.14)	0.01		
Sigmoid, Rectosigmoid, and Rectum	98,581 (20.8%)	2001–2006	3.35 * (0.75 to 10.42)	0.01	−0.22 (−0.76 to 0.42)	0.42
		2006–2021	−1.38 * (−2.29 to −0.83)	<0.001		
Late-Stage Tumors						
Cecum, Ascending Colon, and Hepatic Flexure	67,754 (14.3%)	2001–2009	−1.34 * (−3.23 to −0.56)	<0.001	−0.10 (−0.38 to 0.17)	0.42
		2009–2021	0.73 * (0.28 to 1.60)	0.001		
Transverse Colon	16,561 (3.5%)	2001–2012	−0.64 (−1.88 to 0.13)	0.10	0.97 * (0.58 to 1.37)	<0.001
		2012–2021	2.97 * (1.98 to 4.79)	<0.001		
Splenic Flexure and Descending Colon	23,463 (4.9%)	2001–2013	−0.06 (−0.78 to 0.42)	0.76	0.99 * (0.72 to 1.27)	<0.001
		2013–2021	2.59 * (1.77 to 3.98)	<0.001		
Sigmoid, Rectosigmoid, and Rectum	189,847 (40.0%)	2001–2010	1.34 * (0.55 to 1.88)	0.007	2.44 * (2.26 to 2.64)	<0.001
		2010–2021	3.35 * (3.01 to 3.87)	<0.001		

**Table 3:** Time trends of EO-CRC mortality rates in different age cohorts categorized by tumor stage at diagnosis. ^a^ Death numbers followed by their percentages from the total deaths from EO-CRC. ^b^ Tests whether age-specific trends were identical. A significant *p*-value indicates that the trends were not identical. ^c^ Tests whether age-specific trends were parallel. A significant *p*-value indicates that the trends were not parallel. * Implies statistical significance. ^ There were insufficient sample sizes for at least one calendar year, which hindered the estimation of a trend.

Age Cohort (Years)	Early-Onset CRC Deaths: NCHS (N = 147,026) ^a^ SEER (N = 47,308) ^a^	Trends			Age-Specific AAPC Difference (95% CI)	Pairwise Comparison *p*-Values		
		Time Period	APC (95% CI)	AAPC (95% CI)		Age-Specific AAPC Difference	Test of Coincidence ^b^	Test of Parallelism ^c^
All Stages (CDC’s NCHS Database)								
20–54 years	147,026 (100%)	2000–2005	−1.56 * (−3.52 to −0.56)	0.31 * (0.17 to 0.47)	-			
		2005–2022	0.87 * (0.70 to 1.07)					
20–44 years	39,746 (27.0%)	2000–2022	0.93 * (0.74 to 1.13)	0.93 * (0.74 to 1.13)	0.85 * (0.49 to 1.21)	<0.001	<0.001	<0.001
45–54 years	107,280 (73.0%)	2000–2005	−2.19 * (−3.46 to −0.90)	0.09 (−0.22 to 0.40)				
		2005–2022	0.76 * (0.57 to 0.96)					
Early-Stage Tumors (SEER 22 Database)								
20–54 years	3775 (8.0%)	2004–2006	96.21 * (55.58 to 149.24)	11.88 * (10.40 to 14.88)	-			
		2006–2011	12.65 * (7.99 to 17.87)					
		2011–2021	−0.36 (−2.11 to 0.88)					
20–44 years	699 (1.5%)	^			-			
45–54 years	3076 (6.5%)	2004–2006	90.57 * (46.28 to 165.15)	11.38 * (9.28 to 15.57)				
		2006–2011	12.11 * (4.20 to 19.02)					
		2011–2021	−0.29 (−3.44 to 1.39)					
Late-Stage Tumors (SEER 22 Database)								
20–54 years	37,677 (79.6%)	2004–2006	83.90 * (60.38 to 109.49)	10.69 * (9.38 to 12.16)	-			
		2006–2010	8.21 * (3.85 to 13.65)					
		2010–2021	1.76 * (0.02 to 2.53)					
20–44 years	10,325 (21.8%)	2004–2007	58.84 * (36.56 to 84.75)	10.66 * (7.95 to 13.44)	0.52 (−2.65 to 3.68)	0.74	<0.001	0.09
		2007–2021	2.42 * (1.66 to 3.18)					
45–54 years	27,352 (57.8%)	2004–2006	77.50 * (57.44 to 100.12)	10.15 * (8.58 to 11.73)				
		2006–2010	8.30 * (4.83 to 11.88)					
		2010–2021	1.62 * (1.22 to 2.01)					

## Data Availability

The data used in this study are publicly available and can be obtained from the USCS, NCHS, and SEER websites upon obtaining approval from the databases’ officials.

## Supplementary Materials

The following supporting information can be downloaded at: https://www.mdpi.com/article/10.3390/cancers17091500/s1, Table S1: Number of patients and incidence rates for early-onset colorectal cancer in different demographic populations and tumors’ characteristics. Table S2: Number of deaths and mortality rates for early-onset colorectal cancer in different demographic populations. Table S3: Sensitivity Analysis of Early-Onset Colorectal Cancer Incidence Rates in the US Between 2001–2021 Among Different Age Cohorts (Adenocarcinoma tumors only). Table S4: Number of patients and incidence rates for early-onset colorectal cancer including all histopathological subtypes in different demographic populations and tumors’ characteristics. Table S5: Sensitivity Analysis of Early-Onset Colorectal Cancer Incidence Rates Including All Histopathological Subtypes in the US Between 2001–2021 Categorized by Tumor Anatomical Location in Different Age Cohorts (20–44 years and 45–54 years). Table S6: Time-Trends of Early-Onset Colorectal Cancer (CRC) Incidence Rates Including All Histopathological Subtypes in the US Between 2001–2021 Categorized by Tumor Anatomical Location and Stage at Diagnosis. Table S7: Time-Trends of Early-Onset Colorectal Cancer (EO-CRC) Mortality Rates Including All Histopathological Subtypes the US in Different Age Cohorts (20–44 years and 45–54 years) and Categorized by Tumor Stage at Diagnosis. Figure S1: Age and sex distribution of patients who were diagnosed with early-onset colorectal cancer (EO-CRC) in the United States between 2001 and 2021. Figure S2: Age and sex distribution of patients who died from early-onset colorectal cancer (EO-CRC) in the United States between 2000 and 2022. Figure S3: Time-Trends of Early-Onset Colorectal Cancer Among Different Age Cohorts (Adenocarcinoma tumors only). Figure S4: Age and sex distribution of patients who were diagnosed with early-onset colorectal cancer (EO-CRC) including all histopathological subtypes in the United States between 2001 and 2021. Figure S5: Time-Trends and Age-Adjusted Incidence Rates Per 100,000 Population for Early-Onset Colorectal Cancer (CRC) Including All Histopathological Subtypes Categorized by Tumor Anatomical Location in Different Age Cohorts (Patients Aged 45–54 years and Patients Aged 20–44 years). Figure S6: Time-Trends and Age-Adjusted Incidence Rates Per 100,000 Population for Early-Onset Colorectal Cancer (CRC) Including All Histopathological Subtypes in Adults Aged 20–54 years Categorized by Tumor Anatomical Location and Stage at Diagnosis. Figure S7: Time-Trends and Age-Adjusted Mortality Rates Per 100,000 Population for Early-Onset Colorectal Cancer (EO-CRC) Including All Histopathological Subtypes in Different Age Cohorts (Patients Aged 45–54 years and Patients Aged 20–44 years) and Categorized by Tumor Stage at Diagnosis (Early-Stage and Late-Stage).
