# Supplementary material for: Rising Incidence and Mortality of Early-Onset Colorectal Cancer in Young Cohorts Associated with Delayed Diagnosis
Source: Cancers (Basel). 2025 Apr 29;17(9):1500. doi: 10.3390/cancers17091500 (PMC12071177; doi:10.3390/cancers17091500)
Supplement: Supplementary file 1 [file cancers-17-01500-s001.zip › cancers-3577632-supplementary.pdf]

Supplementary Methods:
Adenocarcinoma cases were defined using the ICD for Oncology, Third Edition, Site Record ICD-O-3/WHO 2008 codes: 8140, 8141, 8143, 8144, 8210, 8211, 8213, 8220, 8221, 8255, 8260–8263, 8310, 8323, 8440, 8460, 8470, 8472, 8480–8482, 8570, 8574, and 8576. This histopathological identification was done in the USCS and SEER 22 databases.

Supplementary Data:
Supplementary Table S1: Number of patients and incidence rates for early-onset colorectal cancer in different demographic populations and tumors' characteristics.

**Variable**	Number of Patients (%)	Age-Adjusted Incidence Rate (per 100,000 population)
**Sex**		
Males	262,075 (55.2%)	16.5
Females	212,526 (44.8%)	13.3
**Age**		
20-44 years	129,938 (27.4%)	6.3
45-54 years	344,663 (72.6%)	37.9
**Race/Ethnicity**		
Non-Hispanic White	319,525 (67.3%)	15.0
Non-Hispanic Black	69,555 (14.7%)	17.6
Hispanic	55,206 (11.6%)	12.2
Non-Hispanic Asian/Pacific Islander	22,276 (4.7%)	12.4
Non-Hispanic American Indian/Alaska Native	4,309 (0.9%)	17
**Tumor Anatomical Location**		
Right Sided Colon	100,517 (21.2%)	3.1
Transverse Colon	25,027 (5.3%)	0.8
Left Sided Colon	35,260 (7.4%)	1.1
Proximal Colon	298,417 (62.9%)	9.3
**Stage At Diagnosis**		
Early Stage	150,950 (31.8%)	4.7
Late Stage	308,216 (64.9%)	9.7
**United States Geographical Region**		
West	98,899 (20.8%)	13.5
Midwest	101,815 (21.5%)	14.8
Northeast	86,855 (18.3%)	14.6
South	187,002 (39.4%)	15.9

Supplementary Table S2: Number of deaths and mortality rates for early-onset colorectal cancer in different demographic populations.

**Variable**	Number of Patients (%)	Age-Adjusted Mortality Rate (per 100,000 population)
**Sex**		
Males	83,173 (56.6%)	4.8
Females	63,853 (43.4%)	3.6
**Age**		
20-44 years	39,746 (27%)	1.8
45-54 years	107,280 (73%)	10.8
**Race/Ethnicity**		
Non-Hispanic White	96,841 (65.9%)	4.1
Non-Hispanic Black	27,284 (18.6%)	6.3
Hispanic	15,291 (10.4%)	3.1
Non-Hispanic Asian/Pacific Islander	6,035 (4.1%)	3.0
Non-Hispanic American Indian/Alaska Native	1,258 (0.9%)	4.5
**Stage At Diagnosis #**		
Early Stage	3,528 (7.5%)	0.3
Late Stage	37,472 (79.2%)	3.3
**United States Geographical Region**		
West	29,872 (20.3%)	3.7
Midwest	31,121 (21.2%)	4.1
Northeast	24,537 (16.7%)	3.8
South	61,496 (41.8%)	4.8

Stage at diagnosis data for early-onset colorectal adenocarcinoma were calculated using incidence-based mortality rates from the Surveillance Epidemiology and End Results (SEER) 22 database.

Supplementary Table S3: Sensitivity Analysis of Early-Onset Colorectal Cancer Incidence Rates in the US Between 2001-2021 Among Different Age Cohorts (Adenocarcinoma tumors only).

[a] Data are presented as count numbers followed by percentages of the count numbers from the total cases of early-onset CRC in the database.

[b] Time-trends were computed using Joinpoint Regression Program (v5.1.0.0, NCI) with 3 maximum joinpoints allowed (4-line segments).

* Implies statistical significance.

Age Cohort (years)	Early-onset CRC Number of Patients (N= 474,601)[a]	Trends[b]				
		Time period	APC (95% CI)	P-Value	AAPC (95% CI)	P-Value
20 – 24 years	2,474 (0.5%)	2001 – 2021	-0.48 (-1.31 to 0.33)	0.23	-0.48 (-1.31 to 0.33)	0.23
25 – 29 years	7,403 (1.6%)	2001 – 2021	0.81* (0.26 to 1.39)	0.003	0.81* (0.26 to 1.39)	0.003
30 – 34 years	17,181 (3.6%)	2001 – 2019	1.64* (0.74 to 6.80)	0.02	1.08* (0.57 to 1.98)	<0.001
		2019 – 2021	-3.80 (-9.12 to 1.69)	0.34		
35 – 39 years	35,043 (7.4%)	2001 – 2021	1.59* (1.40 to 1.79)	<0.001	1.59* (1.40 to 1.79)	<0.001
40 – 44 years	67,837 (14.3%)	2001 – 2021	1.42* (1.16 to 1.69)	<0.001	1.42* (1.16 to 1.69)	<0.001
45 – 49 years	125,258 (26.4%)	2001 – 2011	0.43 (-1.09 to 0.94)	0.32	1.13* (0.89 to 1.37)	<0.001
		2011 – 2021	1.84* (1.36 to 3.18)	0.001		
50 – 54 years	219,405 (46.2%)	2001 – 2011	0.24 (-3.53 to 0.70)	0.43	0.48* (0.12 to 0.86)	<0.001
		2011 – 2021	1.21* (0.41 to 4.32)	0.03		

Supplementary Table S4: Number of patients and incidence rates for early-onset colorectal cancer including all histopathological subtypes in different demographic populations and tumors' characteristics.

Variable	Number of Patients (%)	Age-Adjusted Incidence Rate (per 100,000 population)
**Sex**		
Males	298,588 (54.8%)	18.8
Females	246,172 (45.2%)	15.3
**Age**		
20-44 years	150,906 (27.7%)	7.3
45-54 years	393,854 (72.3%)	43.1
**Race/Ethnicity**		
Non-Hispanic White	359,262 (66.0%)	16.8
Non-Hispanic Black	84,488 (15.5%)	21.3
Hispanic	64,405 (11.8%)	14.2
Non-Hispanic Asian/Pacific Islander	26,187 (4.8%)	14.5
Non-Hispanic American Indian/Alaska Native	4,923 (0.9%)	19.4
**Tumor Anatomical Location**		
Right Sided Colon	110,324 (20.3%)	3.4
Transverse Colon	26,357 (4.8%)	0.8
Left Sided Colon	36,803 (6.8%)	1.2
Proximal Colon	347,917 (63.9%)	10.9
**Stage At Diagnosis**		
Early Stage	184,288 (33.8%)	5.7
Late Stage	329,378 (60.5%)	10.4
**United States Geographical Region**		
West	113,682 (20.9%)	15.4
Midwest	116,105 (21.3%)	16.9
Northeast	99,898 (18.4%)	16.8
South	215,075 (39.5%)	18.2

Supplementary Table S5: Sensitivity Analysis of Early-Onset Colorectal Cancer Incidence Rates Including All Histopathological Subtypes in the US Between 2001-2021 Categorized by Tumor Anatomical Location in Different Age Cohorts (20-44 years and 45-54 years).
[a] Data are presented as count numbers followed by percentages of the count numbers from the total cases of early-onset CRC in the database.
[b] Time-trends were computed using Joinpoint Regression Program (v5.1.0.0, NCI) with 3 maximum joinpoints allowed (4-line segments).
[c] A positive value indicates a greater AAPC in adults aged 20 – 44 years compared to adults aged 45 – 55 years.
[d] Tests whether age-specific trends were identical. A significant P-value indicates that the trends were not identical (i.e., they had different incidence rates and coincidence was rejected).
[e] Tests whether age-specific trends were parallel. A significant P-value indicates that the trends were not parallel (i.e., parallelism was rejected).
* Implies statistical significance.

Age Cohort (years)	Early-onset CRC Number of Patients (N= 544,760)[a]	Trends[b]			Age-specific AAPC difference[c] (95% CI)	Pairwise comparison P-values		
		Time period	APC (95% CI)	AAPC (95% CI)		Age-specific AAPC difference	Test of Coincidence[d]	Test of Parallelism[e]
				**All Anatomical Locations**				
20 – 44 years	150,906 (27.7%)	2001 – 2006	2.29* (1.19 to 3.40)	1.39* (1.10 to 1.68)	0.55* (0.04 to 1.06)	0.03	<0.001	0.001
		2006 – 2021	1.09* (0.90 to 1.29)					
45 – 54 years	393,854 (72.3%)	2001 – 2011	0.31 (-0.35 to 0.97)	0.84* (0.42 to 1.26)				
		2011 – 2021	1.37* (0.74 to 2.01)					
				**Cecum, Ascending Colon, and Hepatic Flexure**				
20 – 44 years	28,935 (5.3%)	2001 – 2021	-0.02 (-0.31 to 0.27)	-0.02 (-0.31 to 0.27)	0.30 (-0.03 to 0.64)	0.07	<0.001	0.05
45 – 54 years	81,389 (14.9%)	2001 – 2021	-0.32* (-0.53 to -0.10)	-0.32* (-0.53 to -0.10)				
				**Transverse Colon**				
20 – 44 years	7,833 (1.4%)	2001 – 2021	1.12* (0.78 to 1.46)	1.12* (0.78 to 1.46)	0.55 (-0.09 to 1.18)	0.09	<0.001	0.03
45 – 54 years	18,524 (3.4%)	2001 – 2013	-0.25 (-0.87 to 0.39)	0.57* (0.03 to 1.12)				
		2013 – 2021	1.81* (0.67 to 2.97)					
				**Splenic Flexure and Descending Colon**				
20 – 44 years	10,918 (2.0%)	2001 – 2021	1.10* (0.74 to 1.45)	1.10* (0.74 to 1.45)	0.56 (-0.14 to 1.26)	0.11	<0.001	0.01
45 – 54 years	25,885 (4.8%)	2001 – 2012	-0.51 (-1.33 to 0.31)	-0.07 (1.15 to 1.72)				
		2012 – 2021	1.83* (0.75 to 2.93)					
				**Sigmoid Colon, Rectosigmoid, and Rectum**				
20 – 44 years	96,173 (17.7%)	2001 – 2006	2.82* (1.50 to 4.16)	1.94* (1.60 to 2.28)	0.55*	0.009	<0.001	0.004
		2006 – 2021	1.64* (1.42 to 1.87)					

45 – 54 years	251,744 (46.2%)	2001 – 2021	1.39* (1.14 to 1.64)	1.39* (1.14 to 1.64)	(0.13 to 0.96)			

Supplementary Table S6: Time-Trends of Early-Onset Colorectal Cancer (CRC) Incidence Rates Including All Histopathological Subtypes in the US Between 2001-2021 Categorized by Tumor Anatomical Location and Stage at Diagnosis.

[a] Data are presented as count numbers followed by percentages of the count numbers from the total cases of early-onset CRC in the database.

[b] Time-trends were computed using Joinpoint Regression Program (v5.1.0.0, NCI) with 3 maximum joinpoints allowed (4-line segments).

* Implies statistical significance.

Tumor Anatomical Location	Early-onset CRC Number of Patients (N= 544,760)[a]	Trends[b]				
		Time period	APC (95% CI)	P-Value	AAPC (95% CI)	P-Value
**All Stages Combined**						
Cecum, Ascending Colon, and Hepatic Flexure	110,324 (20.3%)	2001 – 2021	-0.23 (-0.48 to 0.00)	0.05	-0.23 (-0.48 to 0.00)	0.05
Transverse Colon	26,357 (4.8%)	2001 – 2013	0.20 (-2.08 to 0.72)	0.73	0.75* (0.43 to 1.05)	<0.001
		2013 – 2021	1.59* (0.71 to 4.90)	0.01		
Splenic Flexure and Descending Colon	36,803 (6.8%)	2001 – 2019	0.49* (0.04 to 0.71)	0.04	1.01* (0.64 to 1.22)	<0.001
		2019 – 2021	5.85* (1.17 to 8.13)	<0.001		
Sigmoid, Rectosigmoid, and Rectum	347,917 (63.9%)	2001 – 2021	1.52* (1.31 to 1.74)	<0.001	1.52* (1.31 to 1.74)	<0.001
**Early-Stage Tumors**						
Cecum, Ascending Colon, and Hepatic Flexure	32,707 (6.0%)	2001 – 2007	4.33* (1.75 to 10.64)	<0.001	0.04 (-0.58 to 0.81)	0.91
		2007 – 2021	-1.75* (-2.87 to -0.94)	<0.001		
Transverse Colon	8,246 (1.5%)	2001 – 2006	6.70* (2.85 to 17.63)	<0.001	1.02* (0.27 to 1.83)	0.01
		2006 – 2021	-0.81* (-1.98 to -0.10)	0.02		
Splenic Flexure and Descending Colon	11,337 (2.1%)	2001 – 2015	1.29* (0.64 to 2.32)	0.009	1.05* (0.42 to 1.64)	0.008
		2015 – 2018	-7.92* (-11.61 to -2.11)	0.01		
		2018 – 2021	9.65* (3.33 to 20.55)	0.01		
Sigmoid, Rectosigmoid, and Rectum	128,639 (23.6%)	2001 – 2007	3.36* (1.18 to 10.19)	0.001	0.50 (-0.08 to 1.18)	0.07
		2007 – 2021	-0.70* (-2.01 to -0.09)	0.02		
**Late-Stage Tumors**						
	74,998 (13.8%)	2001 – 2010	-1.01* (-2.71 to -0.38)	0.001	-0.11 (-0.36 to 0.14)	0.34

Cecum, Ascending Colon, and Hepatic Flexure		2010 – 2021	0.62* (0.17 to 1.89)	0.007		
Transverse Colon	17,420 (3.2%)	2001 – 2012	-0.56 (-1.50 to 0.07)	0.07	0.91* (0.59 to 1.25)	<0.001
		2012 – 2021	2.74* (1.93 to 4.12)	<0.001		
Splenic Flexure and Descending Colon	24,386 (4.5%)	2001 – 2012	-0.20 (-1.20 to 0.36)	0.46	0.87* (0.58 to 1.16)	<0.001
		2012 – 2021	2.19* (1.45 to 3.61)	<0.001		
Sigmoid, Rectosigmoid, and Rectum	199,758 (36.7%)	2001 – 2010	1.35* (0.47 to 1.87)	0.009	2.37* (2.19 to 2.56)	<0.001
		2010 – 2021	3.21* (2.87 to 3.75)	<0.001		

Supplementary Table S7: Time-Trends of Early-Onset Colorectal Cancer (EO-CRC) Mortality Rates Including All Histopathological Subtypes the US in Different Age Cohorts (20-44 years and 45-54 years) and Categorized by Tumor Stage at Diagnosis.

[a] Data are presented as death count followed by percentages of the count numbers from the total deaths of EO-CRC in the database.

[b] Time-trends were computed using Joinpoint Regression Program (v5.1.0.0, NCI) with 3 maximum joinpoints allowed (4-line segments).

[c] A positive value indicates a greater AAPC in adults aged 20 – 44 years compared to adults aged 45 – 54 years.

[d] Tests whether age-specific trends were identical. A significant P-value indicates that the trends were not identical (i.e., they had different incidence rates and coincidence was rejected). [e] Tests whether age-specific trends were parallel. A significant P-value indicates that the trends were not parallel (i.e., parallelism was rejected). * Implies statistical significance. ^ There were insufficient sample sizes for at least one calendar year which hindered the estimation of a trend. SEER: Surveillance Epidemiology and End Results.

Age Cohort (years)	Early-onset CRC Deaths (N= 69,798)[a]	Trends[b]			Age-specific AAPC difference[c] (95% CI)	Pairwise comparison P-values		
		Time period	APC (95% CI)	AAPC (95% CI)		Age-specific AAPC difference	Test of Coincidence[d]	Test of Parallelism[e]
**Early-Stage Tumors (SEER 22 Database)**								
20 – 54 years	4,562 (9.8%)	2004 – 2006	96.60* (54.27 to 142.42)	12.17* (10.40 to 14.87)	-			
		2006 – 2011	11.94* (7.28 to 17.97)					
		2011 – 2021	0.37 (-1.39 to 1.61)					
20 – 44 years	912 (2.0%)	^			-			
45 – 54 years	3,650 (7.8%)	2004 – 2006	88.84* (44.13 to 153.72)	11.52* (9.27 to 15.21)				
		2006 – 2011	11.90* (4.37 to 18.79)					
		2011 – 2021	0.20 (-2.91 to 1.78)					
**Late-Stage Tumors (SEER 22 Database)**								
20 – 54 years	42,120 (90.2%)	2004 – 2006	78.85* (60.18 to 98.99)	10.05* (8.85 to 11.23)	-			
		2006 – 2010	7.49* (3.78 to 12.17)					
		2010 – 2021	1.61* (0.07 to 2.29)					
20 – 44 years	11,797 (25.3%)	2004 – 2006	88.19* (52.00 to 133.00)	11.06* (7.92 to 14.29)	1.48 (-1.95 to 4.90)	0.39	<0.001	0.08
		2006 – 2009	9.79 (-2.15 to 23.20)					
		2009 – 2021	2.00* (1.39 to 2.62)					
45 – 54 years	30,323 (64.9%)	2004 – 2006	73.25* (57.43 to 90.67)	9.58* (8.33 to 10.85)				
		2006 – 2010	7.72* (4.88 to 10.64)					
		2010 – 2021	1.45* (1.12 to 1.78)					

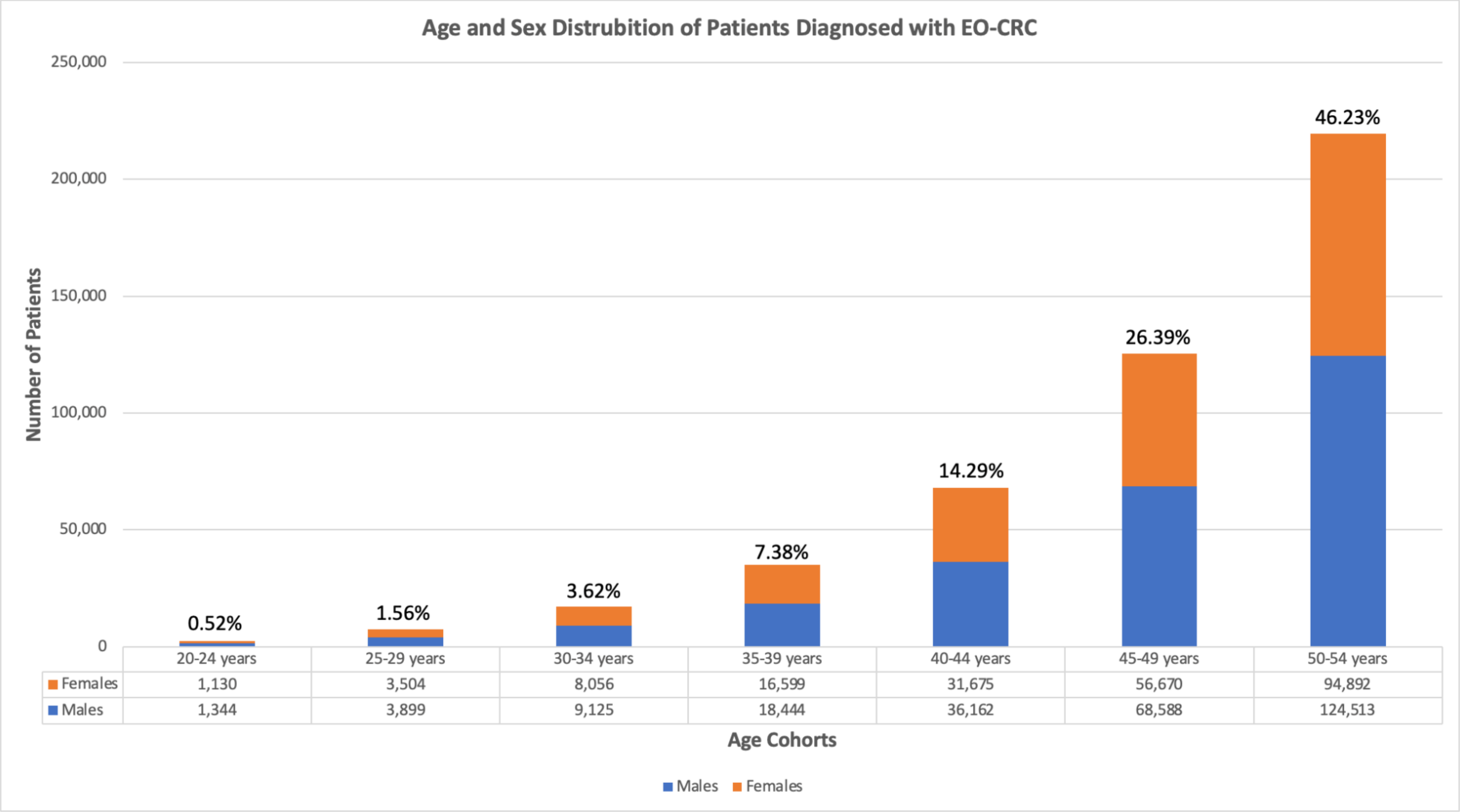

Supplementary Figure S1: Age and sex distribution of patients who were diagnosed with early-onset colorectal cancer (EO-CRC) in the United States between 2001 and 2021.

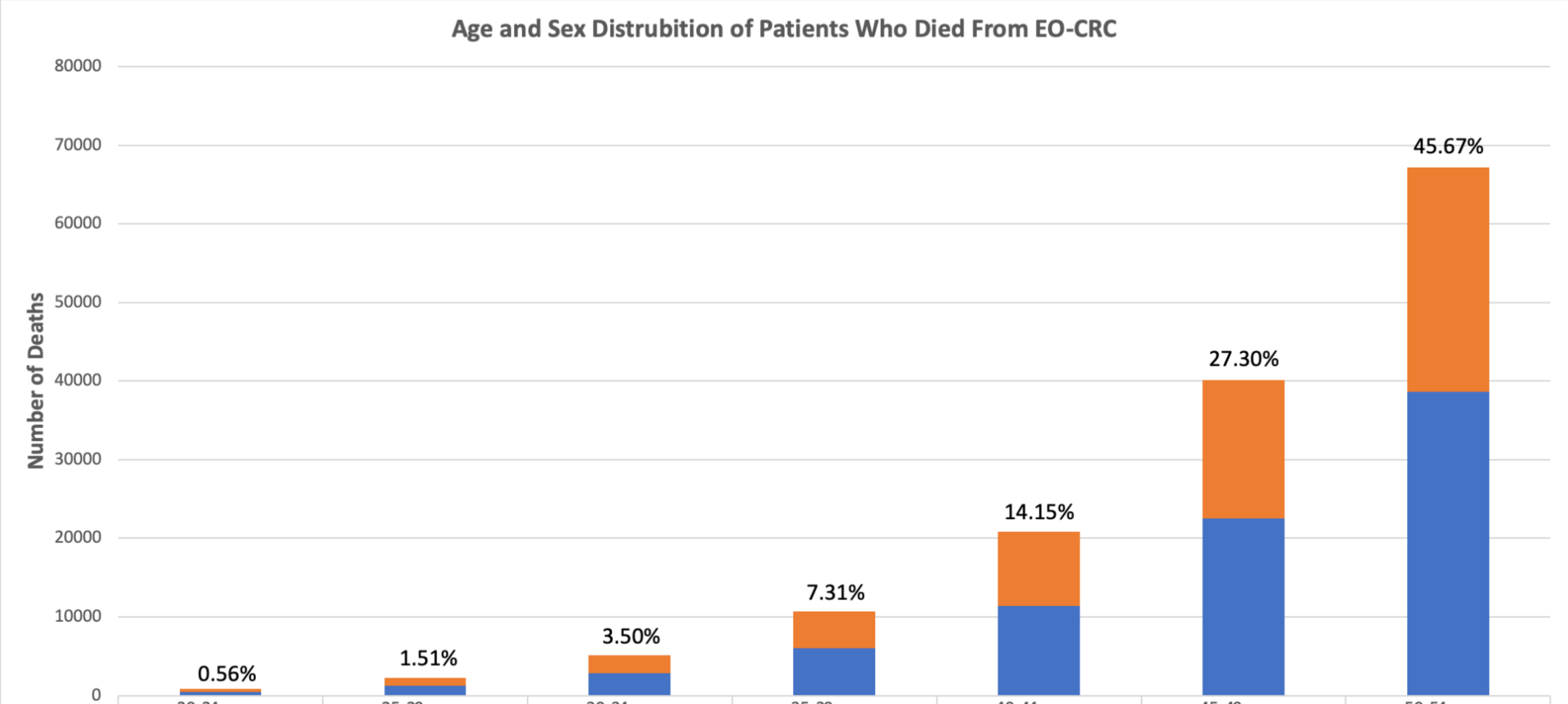

	20-24 years	25-29 years	30-34 years	35-39 years	40-44 years	45-49 years	50-54 years
Females	344	960	2,325	4,768	9,371	17,612	28,473
Males	481	1,265	2,817	5,980	11,435	22,526	38,669

Supplementary Figure S2: Age and sex distribution of patients who died from early-onset colorectal cancer (EO-CRC) in the United States between 2000 and 2022.

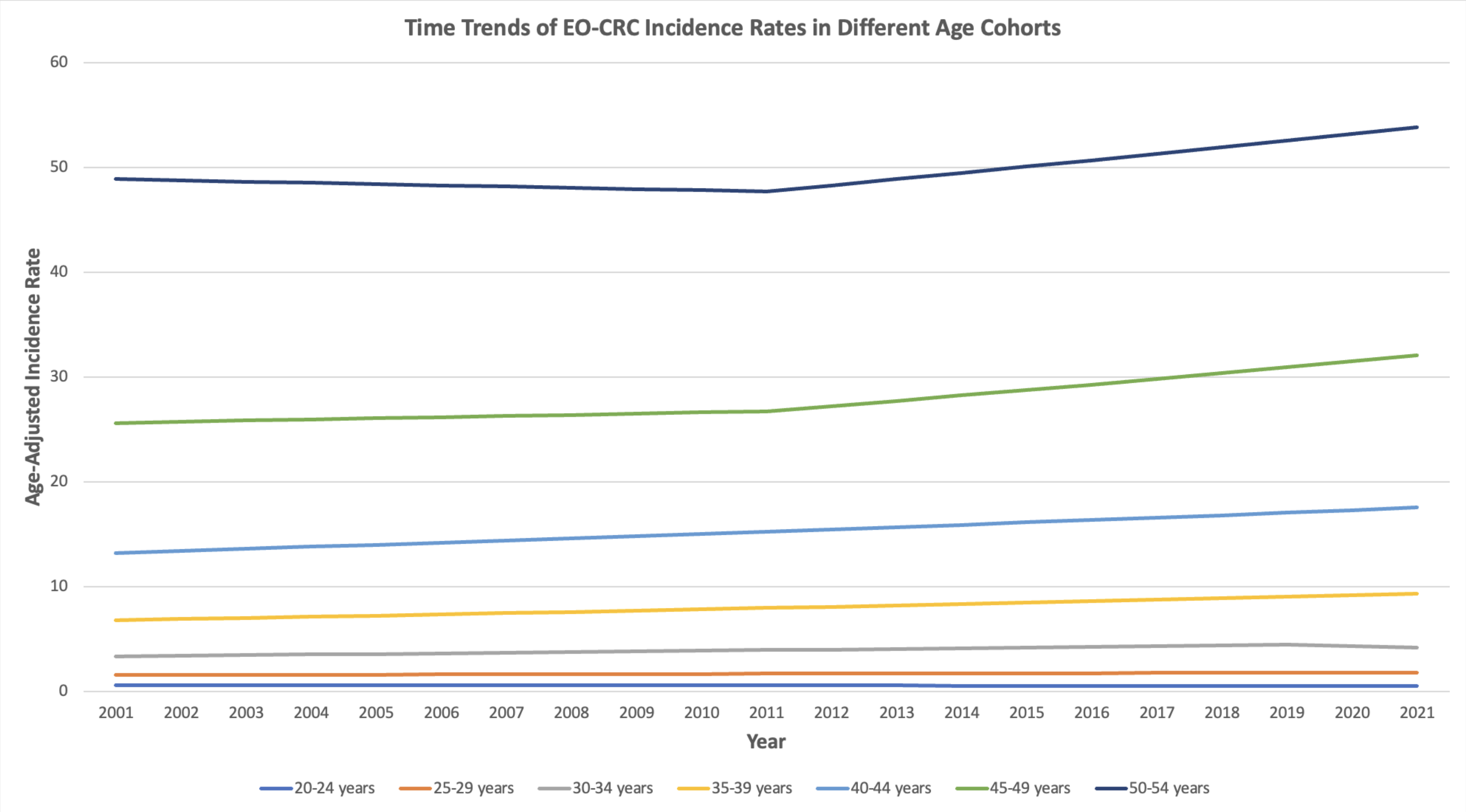

Supplementary Figure S3: Time-Trends of Early-Onset Colorectal Cancer Among Different Age Cohorts (Adenocarcinoma tumors only).

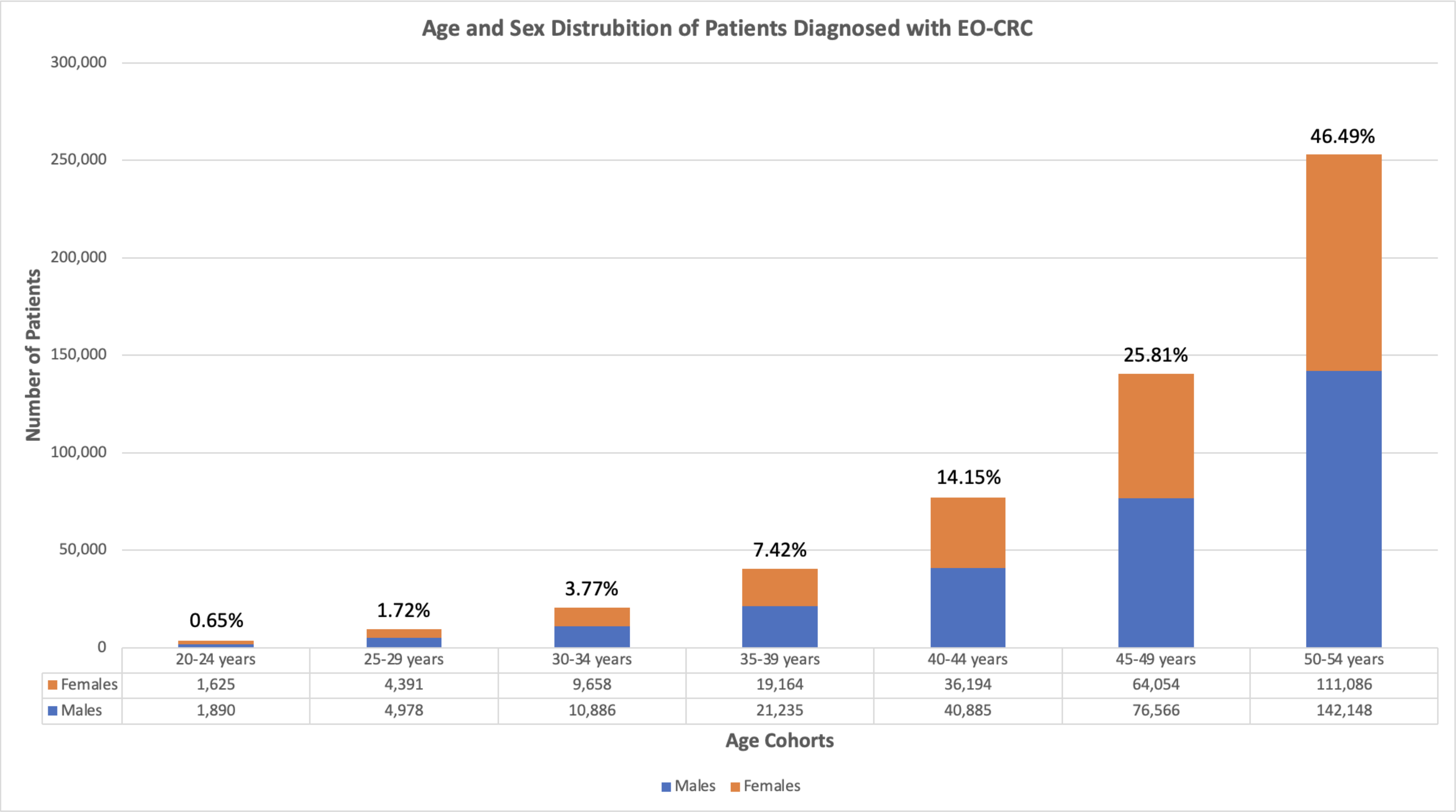

Supplementary Figure S4: Age and sex distribution of patients who were diagnosed with early-onset colorectal cancer (EO-CRC) including all histopathological subtypes in the United States between 2001 and 2021.

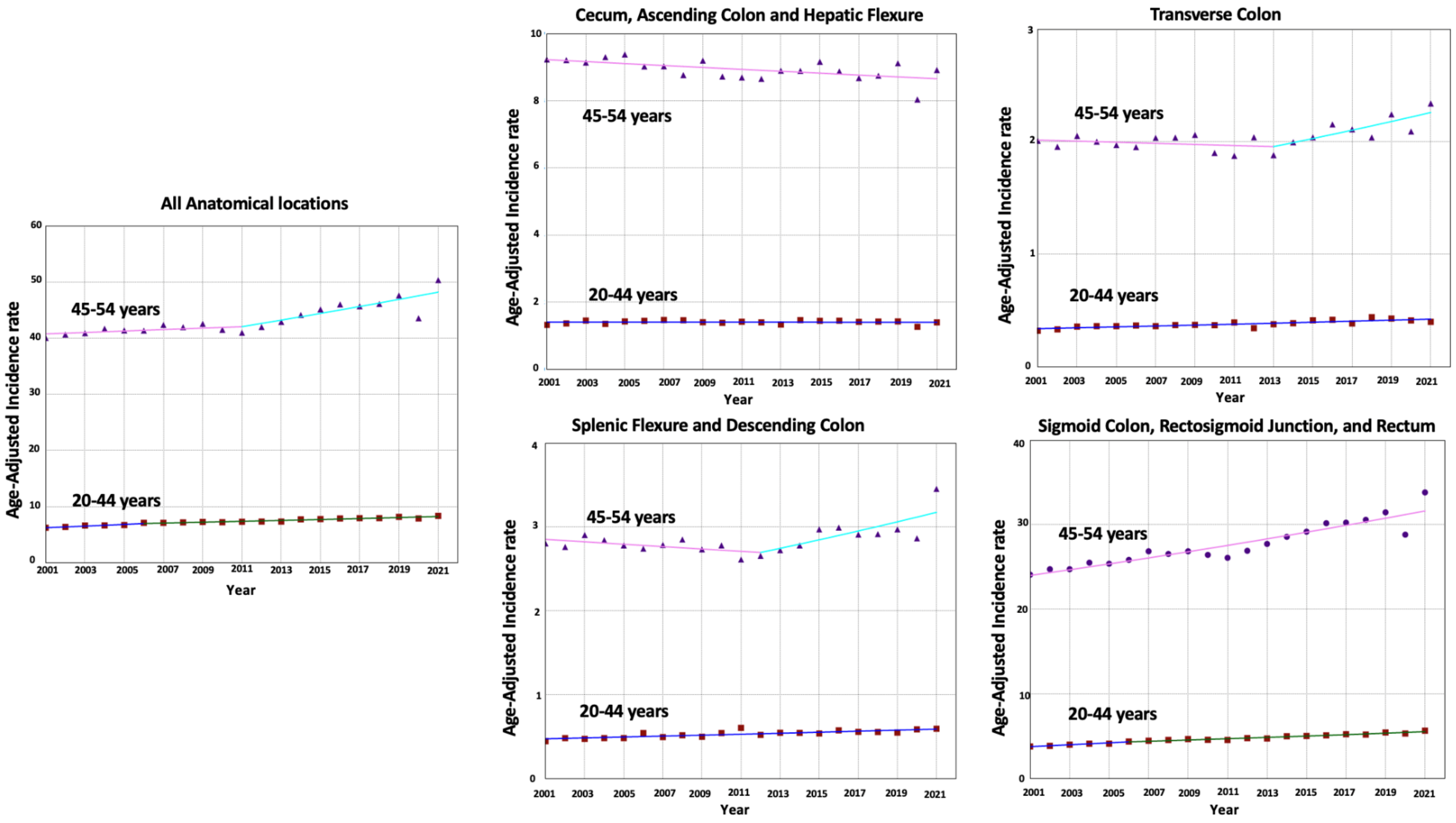

Supplementary Figure S5: Time-Trends and Age-Adjusted Incidence Rates Per 100,000 Population for Early-Onset Colorectal Cancer (CRC) Including All Histopathological Subtypes Categorized by Tumor Anatomical Location in Different Age Cohorts (Patients Aged 45-54 years and Patients Aged 20-44 years).

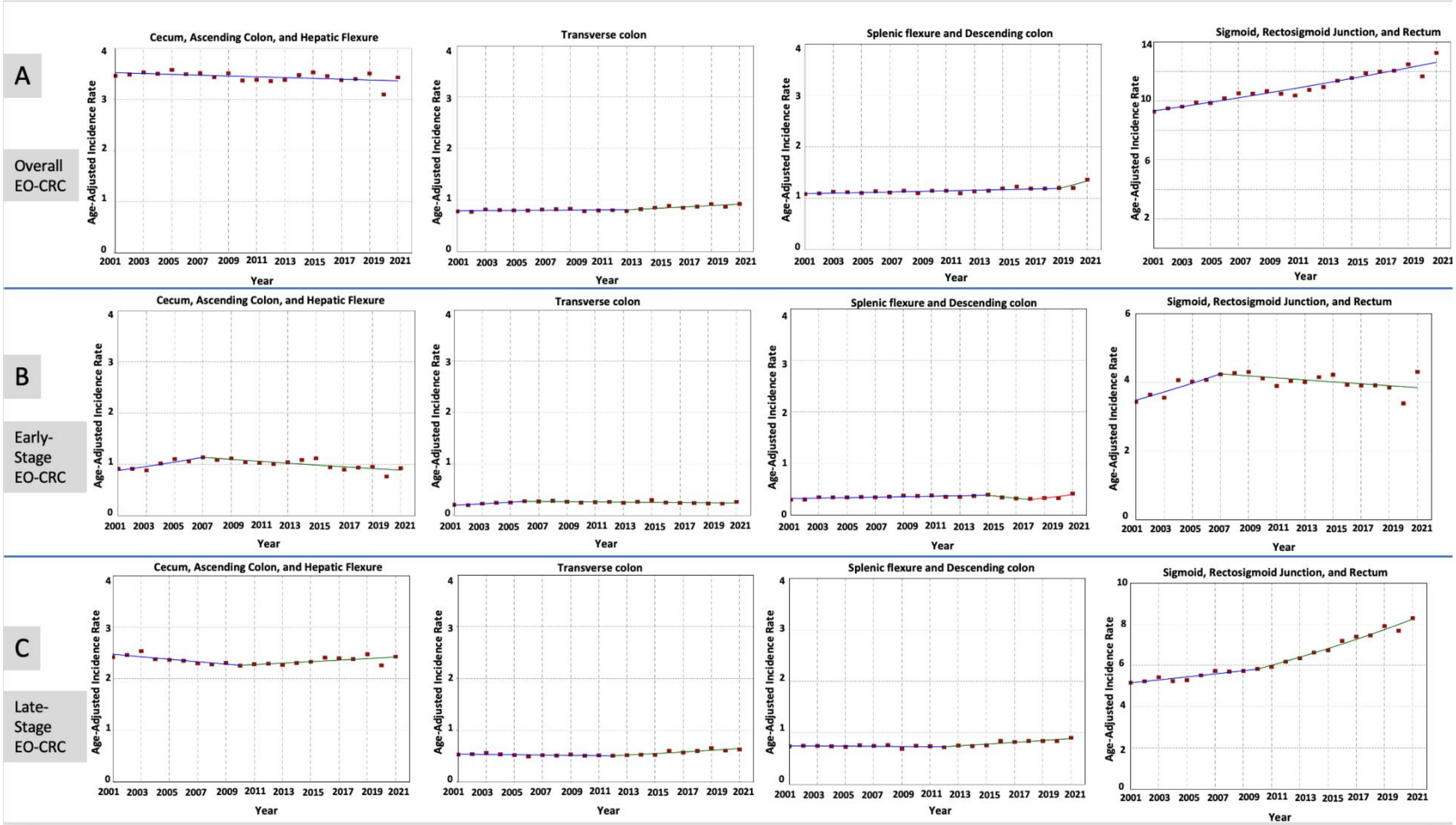

Supplementary Figure S6: Time-Trends and Age-Adjusted Incidence Rates Per 100,000 Population for Early-Onset Colorectal Cancer (CRC) Including All Histopathological Subtypes in Adults Aged 20-54 years Categorized by Tumor Anatomical Location and Stage at Diagnosis.

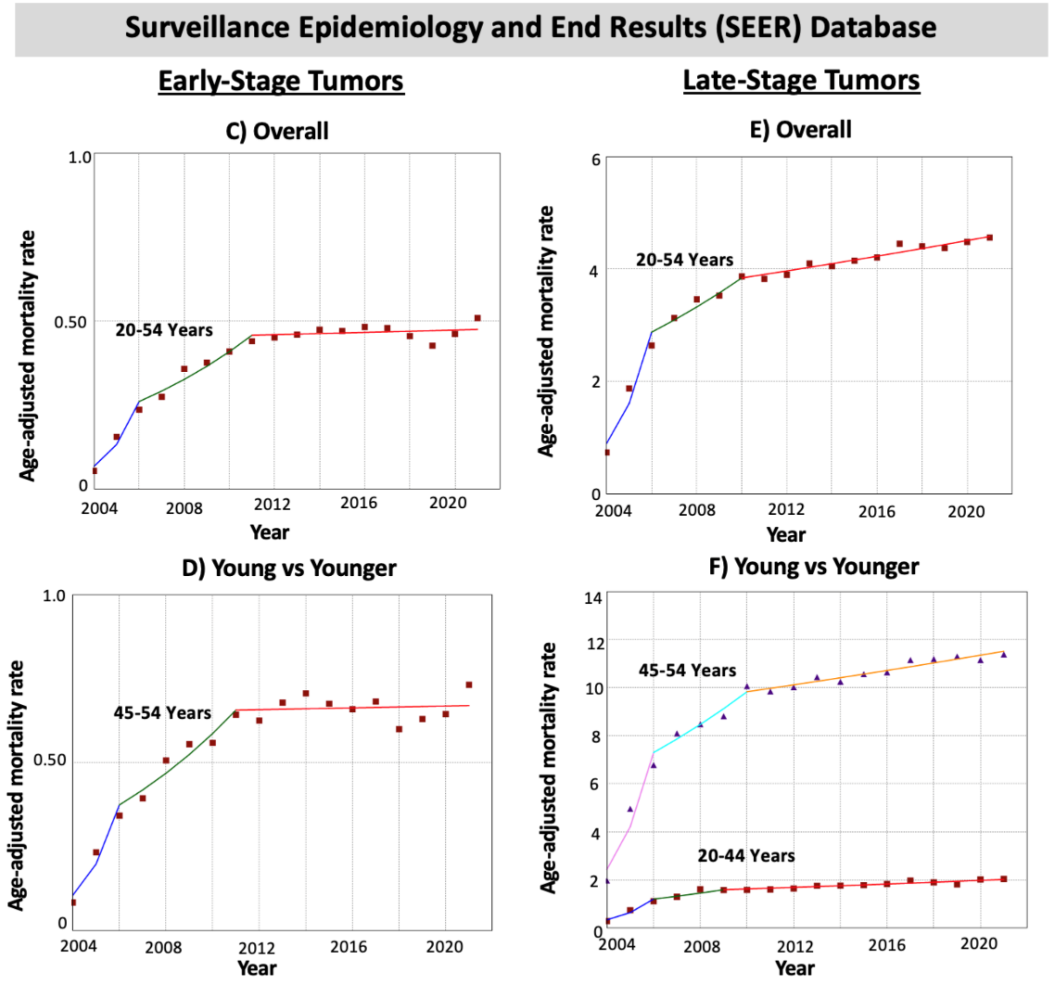

Supplementary Figure S7: Time-Trends and Age-Adjusted Mortality Rates Per 100,000 Population for Early-Onset Colorectal Cancer (EO-CRC) Including All Histopathological Subtypes in Different Age Cohorts (Patients Aged 45-54 years and Patients Aged 20-44 years) and Categorized by Tumor Stage at Diagnosis (Early-Stage and Late-Stage).
